# HOXB7 mediates cisplatin resistance in esophageal squamous cell carcinoma through involvement of DNA damage repair

**DOI:** 10.1111/1759-7714.13142

**Published:** 2019-09-30

**Authors:** Ting Zhou, Hao Fu, Bin Dong, Liang Dai, Yongbo Yang, Wanpu Yan, Luyan Shen

**Affiliations:** ^1^ Key Laboratory of Carcinogenesis and Translational Research (Ministry of Education), Department of Thoracic Surgery I Peking University Cancer Hospital & Institute Beijing China; ^2^ Department of Pathology Peking University Cancer Hospital & Institute Beijing China

**Keywords:** Chemoresistance, ESCC, homeobox gene, NHEJ

## Abstract

**Background:**

DNA damage repair is an important mechanism of platinum resistance. *HOXB7* is one member of HOX family genes, which are essential developmental regulators and frequently dysregulated in cancer. Recently, its relevance in chemotherapy resistance and DNA damage repair has also been addressed. However, little is known regarding the association between HOXB7 and chemotherapy resistance in esophageal squamous cell carcinoma (ESCC).

**Methods:**

The association between HOXB7 expression detected by immunohistochemisty and tumor regression grade (TRG) and long‐term survival was analyzed in 143 ESCC patients who underwent neoadjuvant chemotherapy. CCK8 assay was used to examine the effect of cisplatin in a panel of four ESCC cell lines. A stable cell strain with HOXB7 knockdown of KYSE150 and KYSE450 was established to explore the effect on cisplatin sensitivity. The interaction of HOXB7 with Ku70, Ku80 and DNA‐PKcs was determined by GST‐pull down, coimmunoprecipitation and immunofluorescent colocalization. Finally, we investigated whether disrupting HOXB7 function by a synthetic peptide HXR9 blocking the formation of HOXB7/PBX could enhance cisplatin sensitivity in vitro and in vivo.

**Results:**

High expression of HOXB7 was associated with cisplatin resistance and worse chemotherapy efficacy. HOXB7 knockdown reinforced cisplatin sensitivity. It was identified that HOXB7 interacts with Ku70, Ku80 and DNA‐PKcs. HOXB7 knockdown was related to the downregulation of Ku70, Ku80 and DNA‐PKcs as well as arrested cell cycle in S phase. HOXB7 inhibition by HXR9 had a synergistic effect to improve cisplatin sensitivity.

**Conclusion:**

HOXB7 may be a biomarker for the prediction of chemoresistance of ESCC and serves as a promising therapeutic target.

## Introduction

Esophageal cancer is one of the most common diseases worldwide, with an unsatisfactory overall survival rate. It is mainly comprised of two histopathological types; esophageal adenocarcinoma and esophageal squamous cell carcinoma (ESCC). Currently, surgery plus perioperative chemoradiotherapy dominates the treatment of advanced ESCC, with its efficacy gradually being recognized. A platinum‐based combination regimen is widely used in clinical practice, but chemoresistance is a huge challenge and big obstacle in overall survival improvement because the efficacy improvement is limited to those patients responding to chemotherapy.^1^ Meanwhile, chemoresistance in ESCC attracts less attention compared to other solid tumors (such as lung, breast, and colon cancers, etc) because ESCC occurs mainly in East Asia rather than worldwide. On the other hand, there are few actionable molecular targets for ESCC. In other words, platinum‐based combination chemotherapy is still an essential treatment in ESCC. Thus, at present, exploring the factors influencing platinum response and then targeting it to maintain the response in ESCC must urgently be addressed.

Cisplatin was the first platinum‐based agent shown to have anti‐tumor properties.^2^ Cisplatin‐based combinations remain first‐line therapy for a number of solid tumors including lung, ovarian, and esophageal cancer.^3^ It induces DNA damage through DNA cross‐linking, thus interfering with the fundamental processes of DNA replication and transcription. However, an intrinsic DNA damage repair mechanism is developed in damaged cells to recover genomic integrity and maintain cell viability. These repair mechanisms are not only used by normal cells but also extensively in malignant cells.^4^ If DNA damage is not repaired before mitosis, it leads to cell apoptosis. Therefore, DNA damage repair is an important chemoresistance mechanism through which tumor cells escape from DNA damage induced by cisplatin and thus avoid cell death.

*HOX* genes, which encode a set of transcription factors regulating axial regional specification during embryonic development, are arranged in clusters (*HOXA*, *HOXB*, *HOXC* and *HOXD*) on four separate chromosomes. They have been found dysregulated in many cancers during the last decade and their deregulation influences the malignant phenotype.^5^, ^6^, ^7^, ^8^, ^9^ Besides, the relationship between *HOX* and drug resistance has been reported. HOXA10 was associated with temozolomide resistance through regulation of the homologous recombinant DNA repair pathway in glioblastoma cell lines.^10^ HOXA1 expression was significantly correlated with chemotherapy response. Enforced expression of HOXA1 in SCLC multidrug resistant cell line H69AR led to increased chemosensitivity through increasing cell apoptosis and cell‐cycle arrest.^11^ Knockdown of HOXC6 expression in multidrug resistance cells increased their sensitivity to paclitaxel via its regulation of MDR‐1.^12^ Overexpression of HOXB7 decreased the sensitivity of oral cancer cells to vincristine‐induced apoptosis.^13^ HOXB13 confers Tamoxifen resistance by directly downregulating ERa transcription and inducing IL‐6 expression.^14^ Previously, we found that HOXB7 was overexpressed in ESCC tissues compared with paired noncancerous mucosa, and it promoted oncogenic properties in ESCC cells through affecting cell proliferation, cell cycle and cell apoptosis, and presented negative survival significance in ESCC patients.^15^ Some studies have concentrated on the role of HOXB7 in DNA damage repair and drug resistance. In colon cancer, HOXB7 stimulates DNA repair through interaction with KU70/80 upon etoposide treatment.^16^ Exogenous and endogenous expression of HOXB7 in mammalian epithelial cells enhanced nonhomologous end joining (NHEJ) and DNA damage repair functions through interaction with Ku70, Ku80, DNA‐PKcs.^17^ It is well known that cisplatin exerts cytotoxic effects predominately via nuclear DNA damages, such as single strand break and double strand break (DSB). One of the most important DNA damage repair mechanisms implicated within the context of cisplatin is NHEJ, which repair DSB.^18^ DNA‐dependent protein kinase holoenzyme (Ku70, Ku80, DNA‐PKcs) are the key initiators and responsible for NHEJ. Firstly, the Ku70/80 heterodimer binds and recruits DNA‐PK to sites of DNA strand breaks, where DNA‐PK is activated to remove the DNA lesions, then DNA ligase IV‐XRCC4‐XLF complex rejoins the two DNA ends.^19^

In this study, we found that ESCC cells with higher expression of HOXB7 were more resistant to cisplatin and knockdown of HOXB7 led to increased chemosensitivity and decreased expression of Ku70, Ku80, DNA‐PKcs. We provided evidence to indicate the interaction between HOXB7 and Ku70/Ku80/DNA‐PKcs, which contributes to enhanced NHEJ and cisplatin resistance. Moreover, we utilized a synthesized peptide, HXR9, to disrupt HOX function through blocking the HOX/PBX dimer information and demonstrated that its combination with cisplatin had a synthetic lethality, and achieved better response in vitro and in vivo. It also reinforced the effects of cisplatin to induce DNA damage and cell apoptosis. Thus, we suggested that HOXB7 had a significant impact on chemoresistance in ESCC and could be a biomarker for evaluating chemoresistance and potential target to overcome chemoresistance.

## Methods

### Patients

In January 2000, our research group established a prospective database for esophageal cancer, and collected information on all cases with esophageal cancer to date. After subjects with incomplete paraffin blocks were excluded, 143 ESCC patients treated with neoadjuvant chemotherapy plus surgery by the same surgeon at Peking University Cancer Hospital between January 2000 and December 2011 were recruited for our study including 106 males and 37 females, aged 41–75 years (median age of 59 years), with stage 0–I (*n* = 30), II (*n* = 66), and III (*n* = 51) diseases according to the criteria of the TNM classification system of malignant tumors (AJCC/ UICC). This study was approved by both the Ethics and the Academic committees of Peking University School of Oncology, and informed consent was obtained from all patients. Platinum‐based two‐drug combination was used in neoadjuvant chemotherapy. The detailed neoadjuvant chemotherapy methods and follow‐up information have been described in our previous study.^20^

### Immunohistochemistry

After routine deparaffinization and hydration, postoperative tissue microarray slides were treated with 3% hydrogen peroxide and then heated in citrate buffer (pH6.0) for antigen retrieval. The HOXB7 antigen‐antibody reaction took place overnight at 4°C, following goat serum blocking. The primary antibodies used were anti‐HOXB7 (Abnova, H00003217‐M03 at 3:10000). Dako REAL EnVision Detection System, Peroxisase/DAB, Rabbit/Mouse (K5007) was used as the secondary antibody. The tissue section confirmed as positive staining in our previous study^15^ was used as a positive control, with PBS instead of the primary antibody as a negative control. Immunohistochemical signals were scored by two independent observers. Since tissue microarray was used and the tissue size was too small, definition of the percentage of stained tumor cells was difficult, thus the IHC score was calculated by the staining intensity which was categorized as follows: 0, negative; 1, weak; 2, moderate; and 3, strong. Scores<2 was considered as low‐level expression, whereas scores of ≥2 were considered as high‐level expression.

### Cell lines and cell culture

Human ESCC cell lines KYSE70, KYSE180, KYSE150, KYSE450 were purchased from the Japanese Collection of Research Biosources cell bank (Osaka) and identified by standard STR analysis as well as matching with the American Tissue Culture Collection (ATCC) and Deutsche Sammlung von Mikroorganismen und Zellkulturen GmbH (DSMZ). All cell lines were cultured in RPMI‐1640 medium (Hyclone; GE Healthcare, Logan, UT, USA) supplemented with 10% heat‐inactivated fetal bovine serum and 1% penicillin/streptomycin. The incubator was maintained at 37°C humidified atmosphere containing 5% CO_2_.

### Real‐time PCR

Total RNA was isolated from cell lines using Trizol (Invitrogen, Carlsbad, CA, USA) according to the manufacturer's instructions and reverse‐transcribed using the reverse transcription system (Thermo Fisher Scientific, Waltham, MA, USA). To exclude the possibility of contamination with genomic DNA, we incubated total RNA with RNase‐free DNase at 37°C for 1 hour. The reverse transcription reaction was performed sequentially for 60 minutes at 42°C, and for 5 minutes at 70°C. Real‐time PCR was performed using SYBR Green. PCR runs and fluorescence detection were performed in a Rotor‐Gene 6000 Real‐Time PCR system (Applied Biosystems; Thermo Fisher Scientific, Waltham, MA, USA). We designed cDNA‐specific primers for each gene. The sequences of the real‐time PCR primers for HOXB7 were as follows: forward 5′‐ TATGGGCTCGAGCCGAGTT‐3′ and reverse 5′– GGCCTCGTTTGCGGTCAGT‐3′. The cycling conditions were as follows: 95°C for 10 minutes, followed by 40 cycles of 95°C for 10 seconds, 60°C for 20 seconds, and 72°C for 30 seconds. For each PCR reaction, negative control omission of the reverse transcriptase was done. GAPDH was used as an internal control with the primers as: forward 5′‐ TGCACCACCAACTGCTTAGC‐3′ and reverse 5′‐ GGCATGGACTGTGGTCATGAG‐3′.

### Western blot

The proteins were separated on a 10% SDS‐PAGE gel and transferred to a PVDF membrane followed by western blot analysis, then immunoreacted with anti‐HOXB7 (Abcam, ab51237 at 1:50), anti‐Ku70 (Abcam, ab92450 at 1:1000), anti‐Ku80 (Abcam, ab80592 at 1:1000), anti‐DNA‐PKcs (CST, #5591 at 1:1000), anti‐γ‐H2AX (CST, #9718 at 1:1000) and anti‐GAPDH (ZSGB‐BIO, ZS‐25778 at 1:1000). Goat anti‐rabbit IgG (ZSGB‐BIO, ZB‐2301 at 1:5000) and Goat anti‐mouse IgG (ZSGB‐BIO, ZB‐2305 at 1:5000) were used as the secondary antibodies.

### Cell transfections

ESCC cell lines KYSE150 and KYSE450 were plated (5 × 10^5^ cells) in a six‐well plate and grown overnight to 80% confluence, and then transfected with 100 μL medium containing either short hairpin (lentivirus‐plasmid‐mediated shRNA [short hairpin RNA] sequence as: ACCTGTTCTGTAGCTTTCTGG) targeting HOXB7 gene or scrambled shRNA which constructed into the pGLV‐h1‐GFP‐puro Vector (GenePharma, Shanghai, China) and 8 μg/mL polybrene (Sigma, St. Louis, MO, USA). Stably transfected cells were selected with 2 μg/mL of puromycin (Beyotime Biotechnology, Beijing, China). The knockdown efficiency of HOXB7 was determined by western blot (Fig **S1**).

### CCK8 assay

Cells were plated in 96‐well plates at a density of 5000 cells per well in 100 μL of medium. After treatment with the indicated dose of agents, 100 μL of Cell Counting Kit‐8(CCK8) reagent (Dojindo) was added to each well. After 2 hours of incubation at 37°C, the absorbance per well was measured at 450 nm using a Microplate reader (iMark, Bio‐rad).

### Colony formation assay

Plated cells were treated with the indicated dose of agents at 500 cells/well in six‐well plates. The cells were maintained at 37°C and the completed medium was replaced by a fresh one every three days for the following two weeks. Subsequently obtained colonies were visualized by methanol fixation and 0.1% crystal violet staining. The number of colonies were counted from at least three independent experiments.

### Flow cytometric cell cycle and apoptosis analysis

After treatment of the indicated dose of agents, the cells were fixed in chilled 75% ethylalcohol, and then stained with a PI/RNase staining buffer (BD Biosciences, Franklin Lakes, NJ, USA) for cell cycle analysis. The cells were stained using the Annexin V‐PE or Annexin V‐FITC apoptosis assay kit (Dojindo, Tokyo, Japan) for apoptosis analysis.

### GST‐pulldown

The construct (pGEX‐6P‐1) containing full‐length HOXB7 cDNA was transformed into the BL21 bacterial strain, and single colonies containing the plasmids inoculated into LB with ampicillin (50 μg/ml) and incubate overnight at 37°C. The IPTG(0.1 mM) was added to induce proteins expression when culture grown to OD600 = 0.6. After the IPTG‐induced bacterial solution was added with pulldown lysis buffer (Thermo Scientific), the contained GST‐tagged HOXB7 was immobilized by settled glutathione agarose. Then the protein lysate samples of KYSE150 and KYSE450 was added to the immobilized GST‐tagged HOXB7 and incubate at 4°C at least 1 hour with gentle rocking motion on a rotating platform. Finally, the production of protein: protein was eluted and identified by western blotting. The primary antibodies of anti‐HOXB7, anti‐Ku70, anti‐Ku80 and anti‐DNA‐PKcs were as mentioned above and anti‐GST (Santa Cruz, sc‐374 171 at 1:200).

### Immunofluorescent colocalization

Cells were plated at a density of 1 × 10^5^ cells/well in 12‐well plates with coverslips overnight and then treated with cisplatin (2 μg/mL for KYSE150 and 1 μg/mL for KYSE450) for 24 hours. After being fixed in 3.7% paraformaldehyde for 15 minutes and permeabilized with 0.5% Triton X‐100 for 5 minutes, the coverslips were blocked in 5% normal serum and then incubated in primary antibody dilutions overnight at 4°C. The primary antibodies used were anti‐HOXB7 (Santa Cruz, sc‐81 292 at 1:50), anti‐Ku70(Abcam, ab92450 at 1:100), anti‐Ku80(Abcam, ab80592 at 1:500), anti‐DNA‐PKcs(CST, #38168 at 1:100), anti‐γ‐H2AX(CST, #9718 at 1:400), anti‐p53(CST, #2524 at 1:2000). Then, coverslips were incubated in the appropriate fluorophore‐conjugated secondary antibody dilutions and counterstained with DAPI (Invitrogen, D523 at 300 nmol/L) dilution. The secondary antibodies used were FITC‐conjugated goat anti‐mouse IgG (Invitrogen, F2761 at 1:50) and TRITC‐conjugated goat anti‐rabbit IgG (Invitrogen, T2769 at 1:50). Immunofluorescence was visualized by Zeiss scanning microscope.

### Coimmunoprecipitation

Proteins were extracted by using cold IP lysis buffer (Thermo Fisher Scientific, Waltham, MA, USA) with protease inhibitor cocktail and precleared by the Control Agarose Resin (Thermo Fisher Scientific, 26 150). The HOXB7 antibody (Santa Cruz, sc‐81 292 at 2 μg/mL) was immobilized using AminoLink Plus Coupling Resin. The precleared lysate was then incubated in antibody immobilized resin with gentle mixing overnight at 4°C. After incubation, the resin was eluted using elution buffer (Thermo Fisher Scientific, 21 004). The samples of elution buffer were prepared for western blotting analysis as described above.

### ESCC xenograft models

BALB/c nude mice were raised in the Laboratory Animal Unit of Beijing Cancer Hospital, China. In the preliminary experiment, we predetermined the sample size and estimated that 10 mice for every group were adequate to measure the drug effect. Each 8‐week‐old female Balb/C nude mice was subcutaneously inoculated with 3 × 10^6^ cells of KYSE150 and KYSE450 in 150 μl PBS into the right groin. The sizes of tumors and weight were measured every three days. Tumor volume = ([length] × [width] × [width])/2. When the average tumor volume reached approximately 200 mm^3^, the mice were divided randomly into four groups and received HOXR9 alone, cisplatin alone or a combination of HXR9 and cisplatin treatment, respectively. PBS was used as a control. The tumors were excised after 21 days, and measured by slide caliper for volume and weighed by electronic analytical balance. All experiments were done in accordance with institutional standard guidelines of Peking University Cancer Hospital and Unit of First Affiliated Hospital of PLA General Hospital for animal experiments.

### Synthesis of HXR9 peptide

The HOX/PBX interfering peptide HXR9 was custom synthesized by Sangon Biotech Co. Ltd. (Shanghai). The powdered peptides were dissolved in ddH_2_O to a final concentration of 20 mM and stored at −20°C. HXR9 is an 18‐amino acid peptide consisting of the hexapeptide sequence that can bind with PBX and nine C‐terminal arginine residues (R9) that facilitates it to enter cells. The sequence of peptide is WYPWMKKHHRRRRRRRRR.

### Statistical analysis

SPSS software (version 24.0; IBM SPSS) was used for statistical analysis. Quantitative analysis was performed with the Image J (Version 1.30v; Wayne Rasband). All in vitro experiments were performed at least thrice in triplicates. When the data from different groups were compared, normal analysis and homogeneity of variance were checked first. Comparisons between groups for statistical significance were performed with a two‐tailed unpaired Student's *t*‐test. All values are expressed as mean ± SEM. The relationship between the expression of HOXB7 protein and TRG (tumor regression grade) and clinic‐pathological characteristics was analyzed by Chi‐square test. Univariate survival analysis was carried out using the Kaplan‐Meier method, and subjected to the log rank test. The Cox proportional hazards model with a stepwise procedure was used for multivariate analysis. The variables in the multivariate analysis were age, gender, pT, pN, TRG and HOXB7 expression. *P* < 0.05 was considered significant.

## Results

### HOXB7 increases resistance to cisplatin in ESCC cells

**Figure 1:**
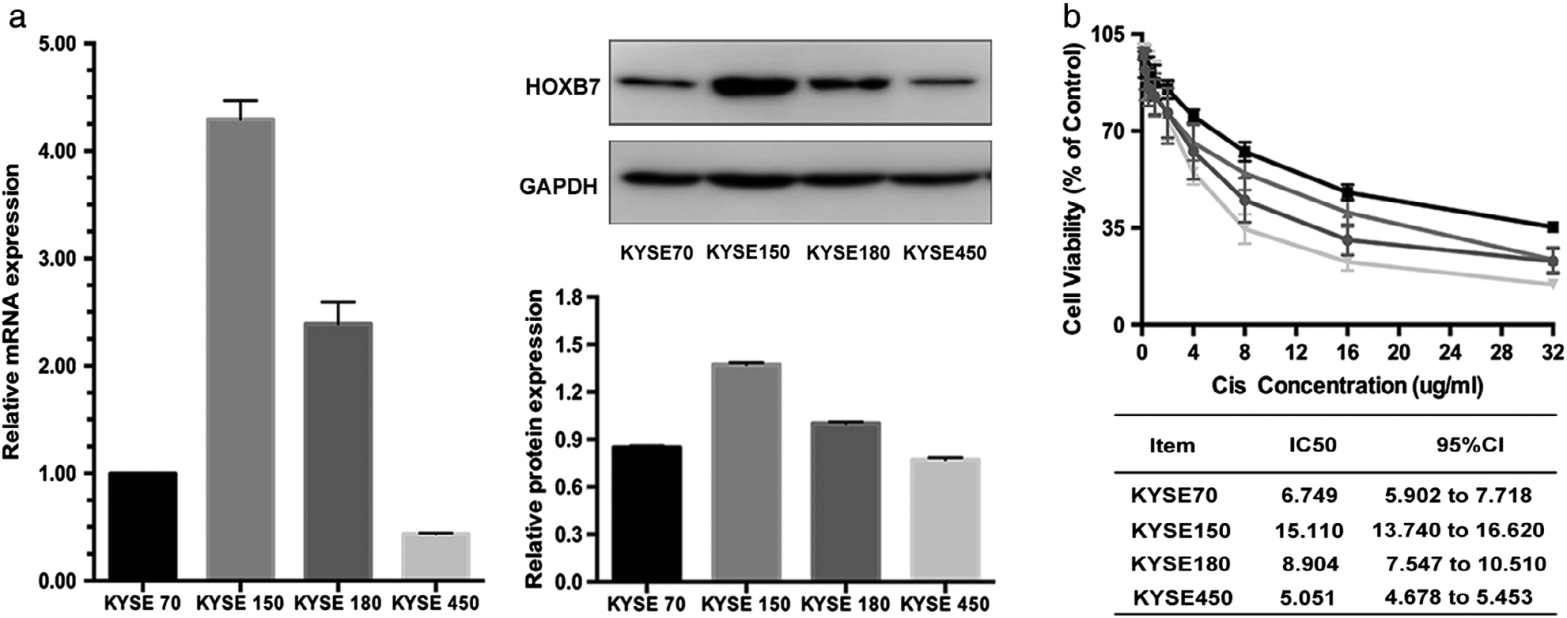
Altered HOXB7 expression in ESCC confers a varied response to cisplatin. (**a**) HOXB7 expression was detected in a panel of four ESCC cell lines at levels of mRNA and protein. According to the relative expression level, they are sorted as KYSE150 > KYSE180 > KYSE70 > KYSE450. (**b**) These four ESCC cell lines were treated with gradient concentration of cisplatin (0.25, 0.5, 1, 2, 4, 8,16,32 μg/mL) for 24 hours, and the cell viability was assessed by using the CCK8 assay. It was shown that KYSE450 was most sensitive, with IC50 = 5.051 μg/mL (95%CI 5.902–7.718), and KYSE 150 was relatively resistant, with IC50 = 15.110 μg/mL (95%CI 13.740–16.620). It was considered that a high expression of HOXB7 was associated with cisplatin resistance. This experiment was performed thrice in triplicates. (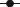) KYSE 70, (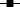) KYSE 150, (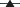) KYSE 180, and (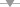) KYSE 450.

To analyze the association between HOXB7 expression and cisplatin sensitivity in ESCC cells, we examined HOXB7 expression on the levels of mRNA and protein in four ESCC cell lines (KYSE150, KYSE180, KYSE70 and KYSE450) (Fig 1a), then CCK‐8 assay was performed to investigate the effect of cisplatin on cell viability. The results showed that cells with lower expression of HOXB7 were relatively sensitive to cisplatin with lower IC50 (Fig 1b), and it was hypothesized that high HOXB7 may be involved in chemoresistance.

**Figure 2:**
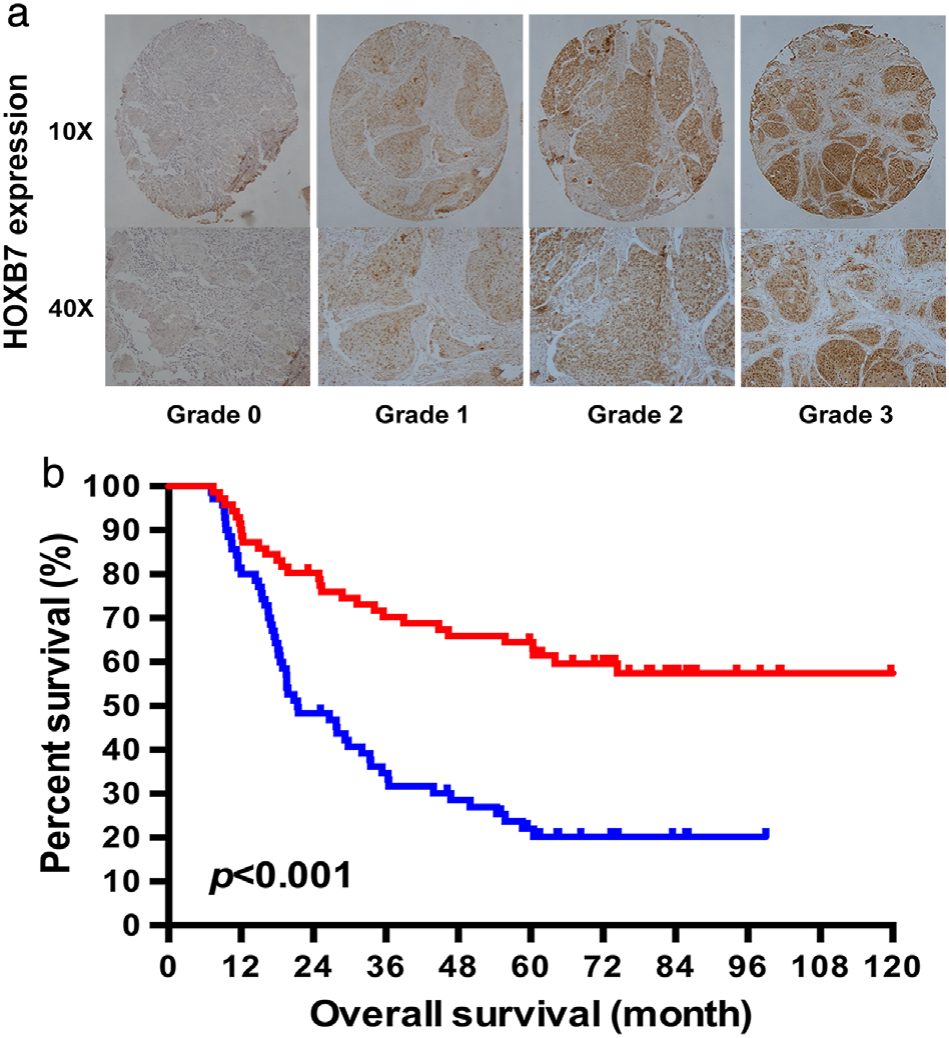
The association between HOXB7 expression and the survival of patients with ESCC who underwent neoadjuvant chemotherapy. (**a**) HOXB7 expression pattern by the intensity of positive tumor cells. 0, Negative; grade 1, weak; grade 2, moderate; grade 3, strong. (**b**) The Kaplan‐Meier survival curve showed that the median survival time (MST) of patients with high HOXB7 expression was significantly shorter than that of patients with low expression. (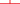) HOXB7 Low expression and (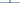) HOXB7 High expression.

We then examined the expression of HOXB7 in post‐operative tumor tissues of patients with ESCC undergoing neoadjuvant chemotherapy, and the relationship between HOXB7 expression with TRG (tumor regression grade) and overall survival was analyzed. The association between HOXB7 expression and clinicopathological characteristics are presented in Table S1. As Fig 2 and Tables S2 and S3 show, patients with higher expression of HOXB7 had worse TRG and poorer prognosis. In other words, patients with higher HOXB7 expression had a worse response to chemotherapy, which led to an inferior outcome. Although chemotherapy may change the expression of genes or proteins probably including HOXB7, which results in less solid conclusion, it is a way of expediency to use post‐operative tissues for analysis since biopsy specimens prior to chemotherapy are often too small and difficult to obtain.

### Downregulation of HOXB7 enhances chemosensitivity

**Figure 3:**
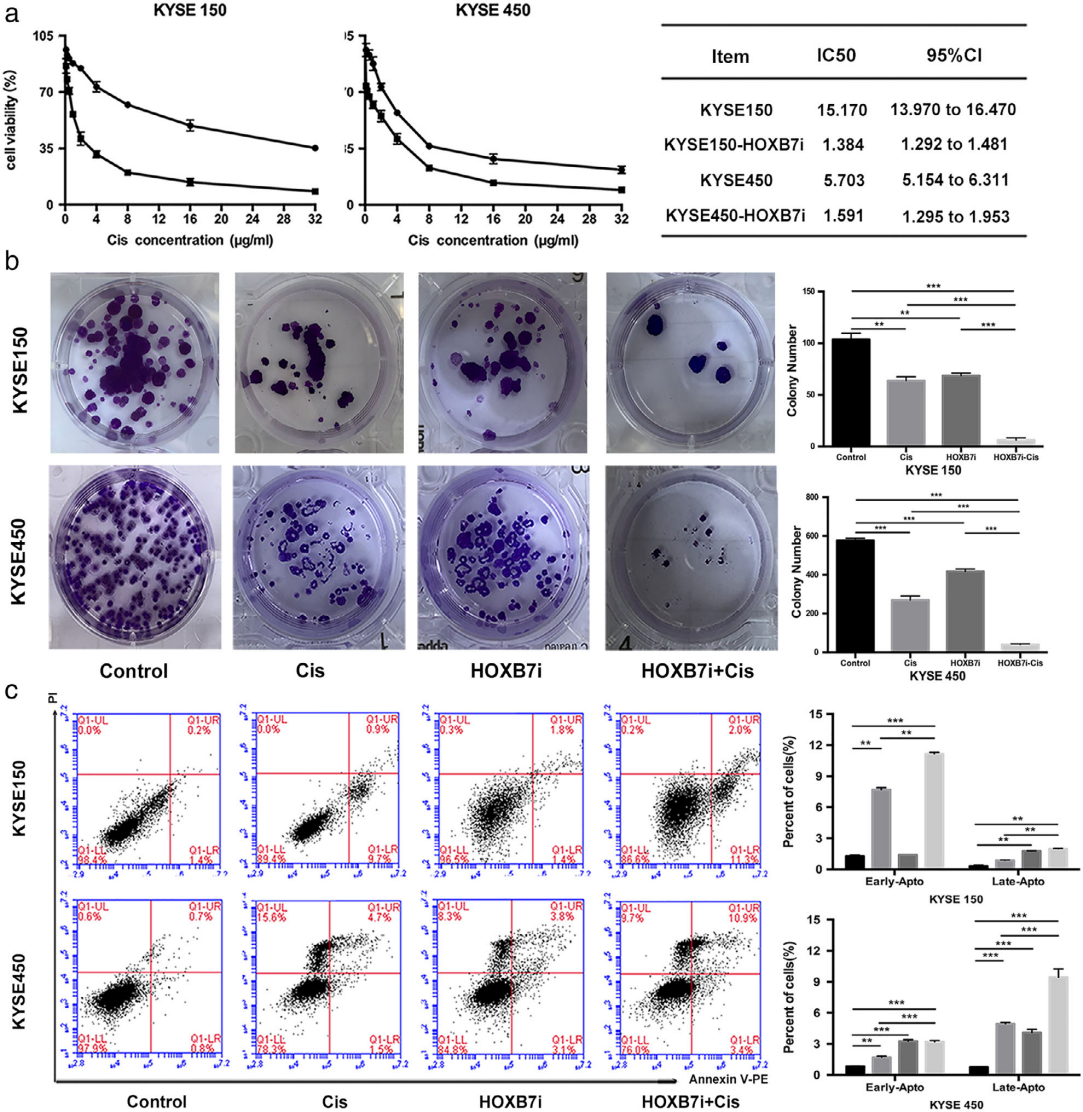
Downregulation of HOXB7 enhances sensitivity to cisplatin. (**a**) We established stable HOXB7‐knockdown cell strains (KYSE450‐HOXB7i, KYSE150‐HOXB7i) to determine the effect of HOXB7 on chemosensitivity. CCK8 assay showed that cell viability of HOXB7‐knockdown cells was reduced with a smaller IC50 value than control cells (1.384 vs. 15.170 for KYSE150, 1.591 vs. 5.703 for KYSE450). (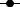) Cis and (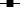) HOXB7i‐Cis (**b**) Colony formation assay showed that there were less colony formation in HOXB7‐knockdown cells upon exposure to cisplatin (2 μg/mL for KYSE150 and 1 μg/mL for KYSE450) than control cells. (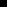) Control, (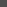) Cis, (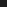) HOXB7i, and (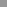) HOXB7i‐Cis. (**c**) HOXB7‐knockdown sensitizes ESCC cells to cisplatin (2 μg/mL for KYSE150 and 1 μg/mL for KYSE450) induced apoptosis. All experiments were performed thrice in triplicates. (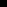) Control, (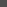) Cis, (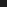) HOXB7i, and (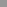) HOXB7i‐Cis.

To determine the effects of HOXB7 expression on the response to cisplatin, we selected KYSE150 and KYSE450 cell lines as subjects because KYSE150 was the most resistant whereas KYSE450 was the most sensitive. We then established stable HOXB7‐knockdown cell strains via RNA inference (named KYSE150‐HOXB7i, KYSE450‐HOXB7i). These cell strains were used to determine the effect of HOXB7 on cell viability, and cell apoptosis after exposure to cisplatin. CCK8 assay showed that treatment for 24 hours with cisplatin resulted in decreased IC50 of HOXB7‐knockdown cells compared with control cells (Fig 3a). This means that knockdown of HOXB7 increases the sensitivity to cisplatin. Colony formation assay also confirmed that the cell viability and cell proliferation in HOXB7‐knockdown cells dramatically decreased upon exposure to low‐dose cisplatin (Fig 3b). Flow cytometry assay showed that cisplatin induced a significant increase in cell apoptosis in HOXB7‐knockdown cells (Fig 3c).

**Figure 4:**
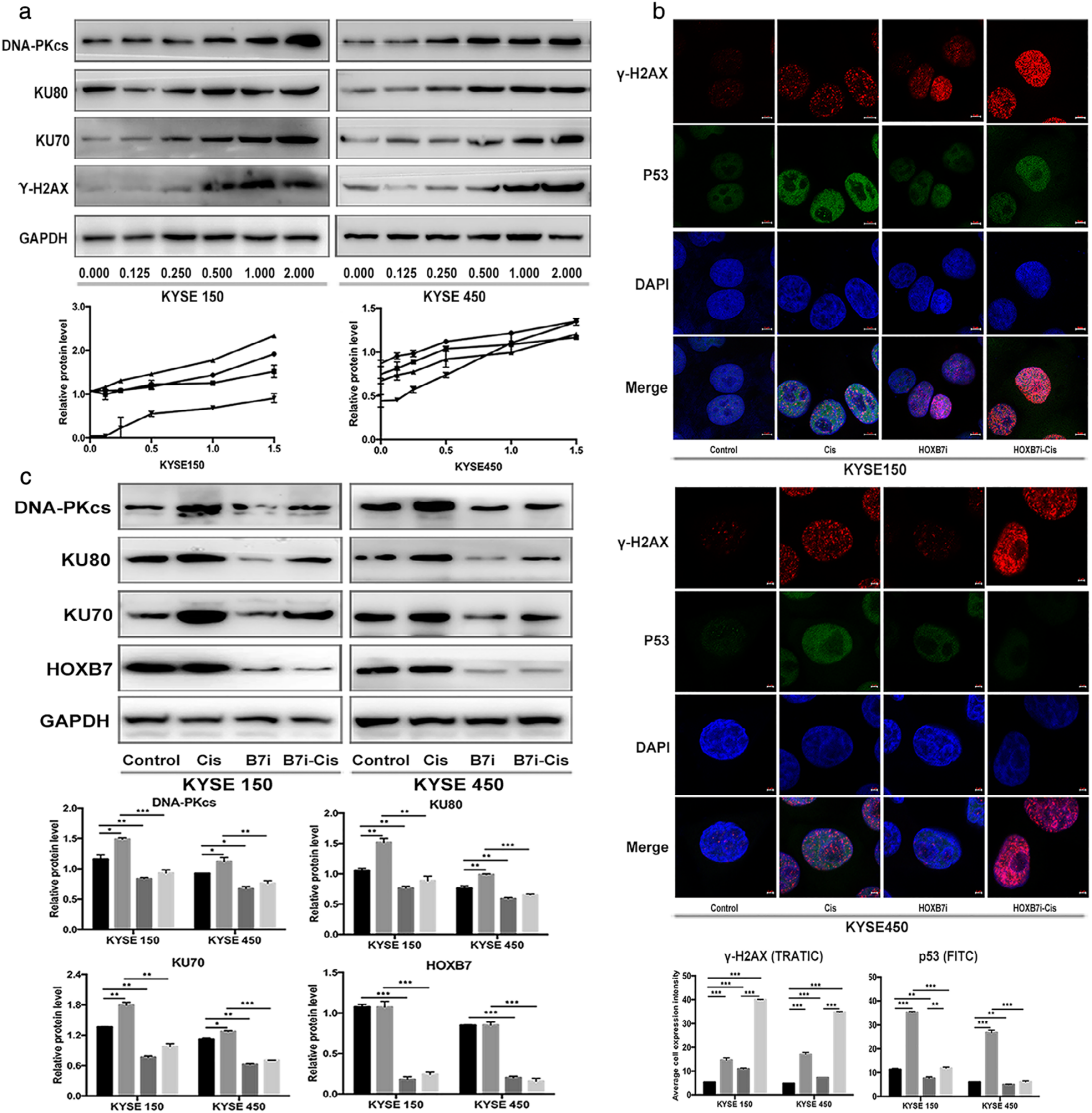
Downregulation of HOXB7 enhances DNA damage effect. (**a**) Cisplatin induces DNA damage and activates DNA damage repair mechanism. As western blotting detected, expression of γ‐H2AX (a surrogate marker for DSB) in KYSE150 and KYSE450 presented a dose‐dependent increase upon exposure to cisplatin (0.125, 0.25, 0.5 1 and 2 μg/mL), and the NHEJ components Ku70, Ku80, DNA‐PKcs were also upregulated in a similar manner. (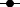) DNA‐PKC, (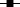) KU80, (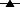) KU70, and (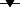) y'‐H2AX. (**b**) HOXB7 knockdown enhanced DNA damage effect and impaired DNA damage response presented by examining fluorescence intensity of γ‐H2AX and p53 in ESCC cells. After knockdown of HOXB7, the γ‐H2AX fluorescence intensity upon exposure to cisplatin (2 μg/mL for KYSE150 and 1 μg/mL for KYSE450) had a stronger amplification than control cells, but p53 fluorescence intensity presented a weaker enhancement than control cells. (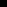) Control, (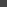) Cis, (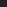) HOXB7i, and (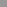) HOXB7i‐Cis. (**c**) In HOXB7‐knockdown cells, the enhancement of Ku70, Ku80, DNA‐PKcs expression upon exposure cisplatin (2 μg/mL for KYSE150 and 1 μg/mL for KYSE450) was less than control cells. (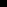) Control, (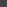) Cis, (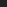) HOXB7i, and (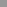) HOXB7i‐Cis.

Cisplatin crosslinks with DNA to form cisplatin‐DNA adducts and induce intra‐ and interstrand crosslinking, leading to DSB. The intrinsic DNA damage response mechanism is then initiated relying on the activation of two major checkpoint pathways. The sequentially activated checkpoint pathway activates the downstream effector such as p53 to upregulate cell cycle checkpoint pathways and then repairs the DNA damage. Previously, we found that γ‐H2AX (well‐accepted surrogate marker of DSB for eukaryotic cells) and p53 fluorescence intensity in ESCC cells amplified after exposure to cisplatin.^20^ In this study, γ‐H2AX expression in KYSE150 and KYSE450 was found to present a dose‐dependent increase after exposure to cisplatin as detected by western blotting, and Ku70, Ku80, DNA‐PKcs, which triggers NHEJ, also exhibited increased expression likewise (Fig 4a). This means that cisplatin causes DNA damage and then sequentially activates the DNA damage repair. However, the increment of γ‐H2AX in HOXB7‐knockdown cells after exposure to cisplatin was significantly stronger than control cells, and p53 fluorescence intensity increment presented in an opposite manner (Fig 4b). Ku70, Ku80, DNA‐PKcs expression was decreased in HOXB7‐knockdown cells but their expression upon exposure to cisplatin in HOXB7‐knockdown cells increased to a lesser extent than that in control cells (Fig 4c). Therefore, it is proposed that HOXB7 knockdown may increase chemosensitivity partially through disrupting DNA damage response.

### Downregulation of HOXB7 induces cell cycle arrest in S phase exposure to cisplatin

**Figure 5:**
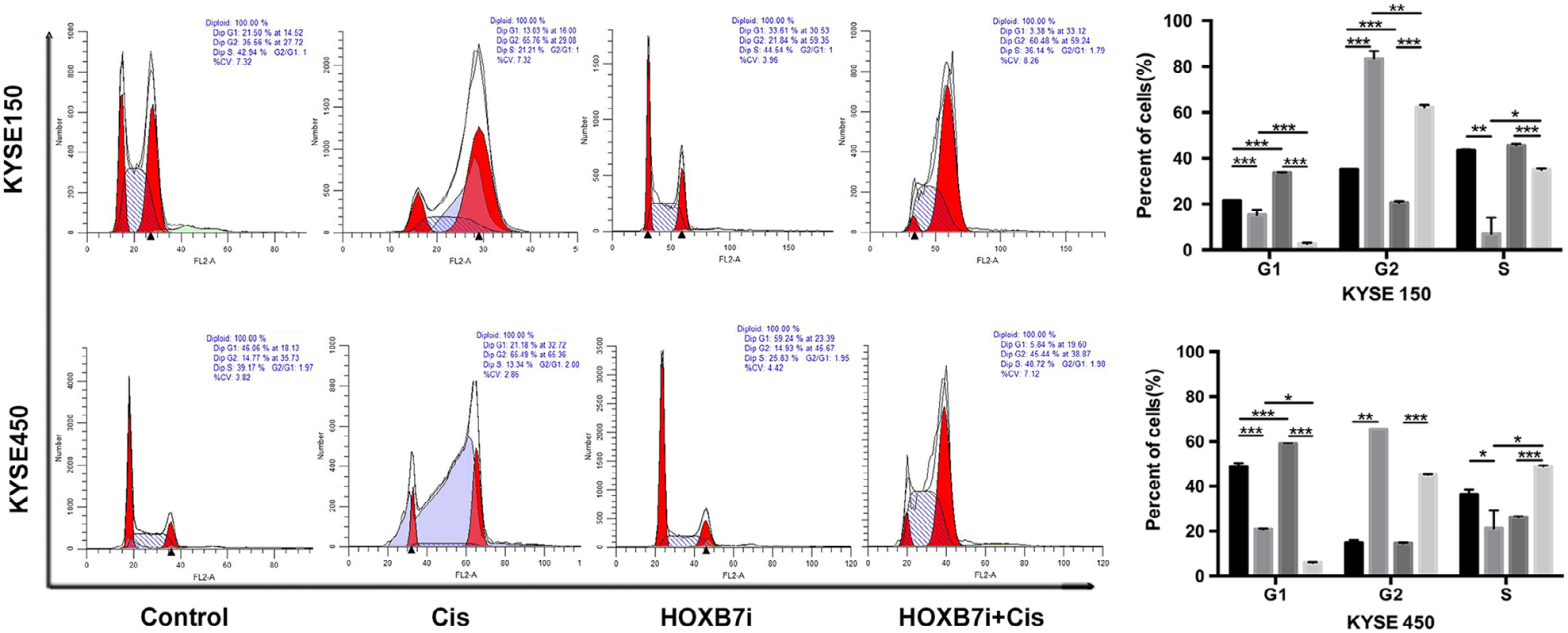
HOXB7 knockdown induces cell cycle arrest in S exposure to cisplatin. Cells were treated with the indicated concentration of cisplatin (2 μg/mL for KYSE150 and 1 μg/mL for KYSE450) for 24 hours and underwent flow cytometry analysis by staining with PI/RNase stain to assess the cell cycle progression. Cell cycle was arrested in phase S for HOXB7‐knockdown cells by cisplatin treatment compared to control cells. This experiment was performed thrice in triplicates. (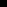) Control, (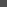) Cis, (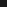) HOXB7i, and (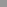) HOXB7i‐Cis.

Previously, it has been shown that knockdown of HOXB7 induced cell cycle arrest in G1/S phase.^15^ We evaluated the impact of HOXB7 on cell cycle when exposed to cisplatin in this study. By flow cytometry analysis, the results showed that knockdown of HOXB7 in KYSE150 and KYSE450 decreased cell cycle arrest in G1 induced by cisplatin but increased cells in S phase (Fig 5). Although cisplatin is considered to lack cell cycle specificity, cytotoxicity is increased with exposure to the drug during S phase.^21^ Thus, we suggested that knockdown of HOXB7 enhanced chemosensitivity through inducing S phase arrest. On the other hand, DSBs are repaired predominately via NHEJ in G1‐phase.^22^ It was implied that knockdown of HOXB7 sensitized cells to cisplatin through decreased G1 arrest, led to declined NHEJ repair activity, which was confirmed by another evidence that NHEJ components Ku70, Ku80 and DNA‐PKcs expression was decreased in HOXB7‐knockdown cells.

### HOXB7 interacts with DNA damage repair protein Ku70/Ku80/DNA‐PKcs in vitro and in vivo

**Figure 6:**
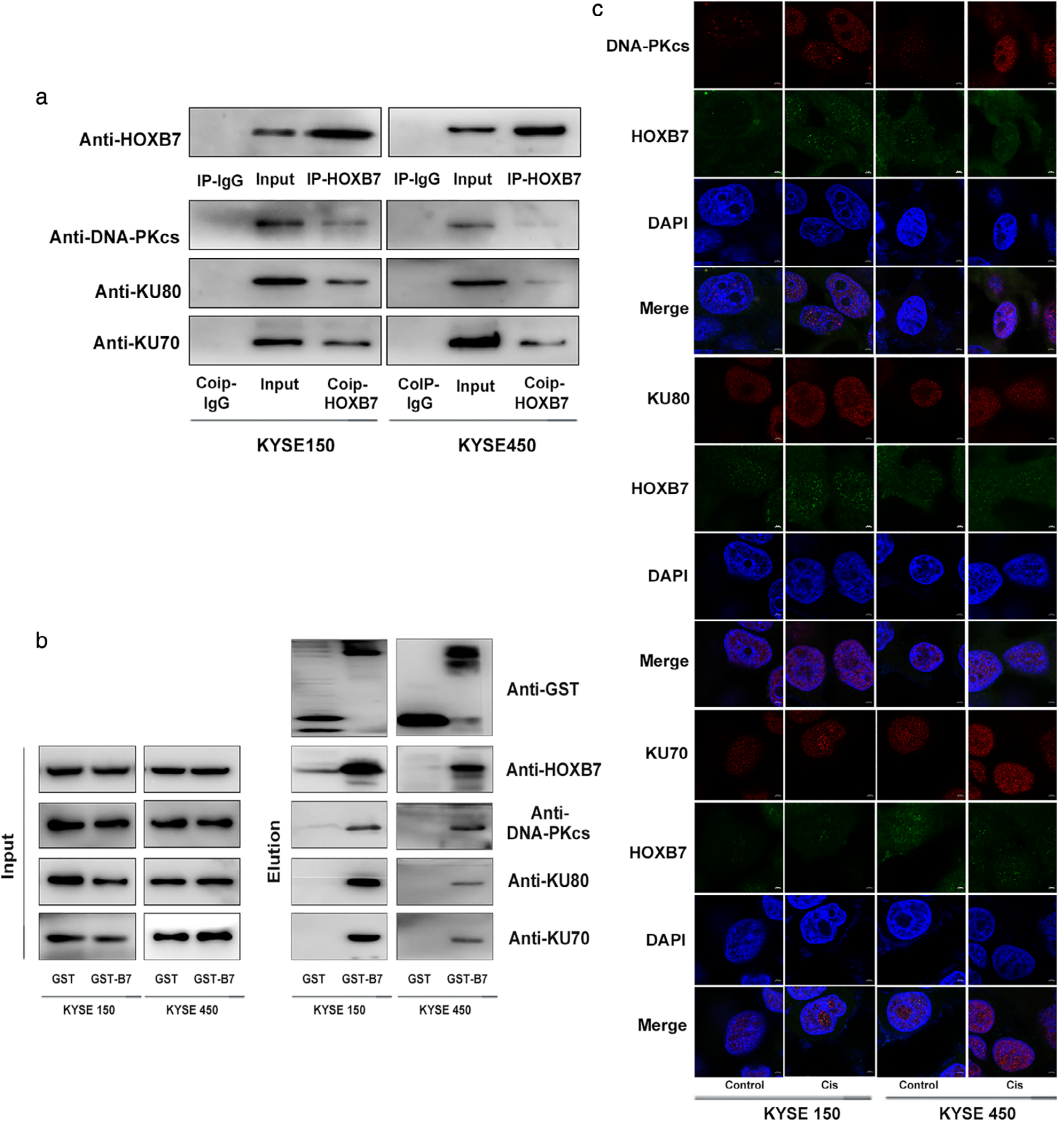
Identification of HOXB7 interacting with Ku70, Ku80 and DNA‐PKcs. (a) Coimmunoprecipitation of HOXB7 with Ku70, Ku80 and DNA‐PKcs in kYSE150 and KYSE450 cells. Protein was extracted and precipitated using anti‐HOXB7 antibody. Precipitates were subjected to western blotting using anti‐Ku70, anti‐Ku80 and anti‐DNA‐PKcs. (**b**) GST‐HOXB7 fusion protein pulldown assay in KYSE150 and KYSE450 cells. (**c**) GST‐HOXB7 fusion constructs were bacterially expressed. KYSE150 and KYSE450 cell lysates were mixed with glutathione agarose containing GST, GST‐HOXB7 construct and probed by western blot with an antibody against Ku70, Ku80 and DNA‐PKcs. (**d**) The colocalization of HOXB7 with Ku70, Ku80, DNA‐PKcs was analyzed by immunofluorescence microscopy. HOXB7 colocalized with Ku70, Ku80, DNA‐PKcs in nuclear, which was more intensive upon exposure to cisplatin (2 μg/mL for KYSE150 and 1 μg/mL for KYSE450).

NHEJ is one of main repair mechanisms for DSB, and the significant role of HOXB7 involving in NHEJ has been demonstrated. It has been shown that HOXB7 enhanced NHEJ and DNA damage repair functions via interaction with Ku70/Ku80/DNA‐PKcs.^16^, ^17^ Therefore, we focused our efforts on investigating if the interaction of HOXB7 with Ku70/Ku80/DNA‐PKcs exists in ESCC cells. Firstly, we attempted to identify the interacting between HOXB7 and Ku70, Ku80, and DNA‐PKcs through coimmunoprecipitation. The results indicated HOXB7 interacting with Ku70, Ku80, DNA‐PKcs (Fig 6a). To further test whether HOXB7 interacts directly with Ku70, Ku80, and DNA‐PKcs, bacterial expressed GST‐HOXB7 recombination protein was bound with high affinity to glutathione agarose, followed by “pulldown” of interacting proteins via interaction of agarose‐protein complexes with the cell lysate of KYSE150, KYSE450. The complexes were then subjected to western blotting using anti‐Ku70, anti‐Ku80, and anti‐DNA‐PKcs. As shown in Fig 6b, the interaction between HOXB7 and Ku70, Ku80, DNA‐PKcs was present.

Moreover, we observed the cellular localization of HOXB7 and Ku70/Ku80/DNA‐PKcs to confirm their proximity in space using immunofluorescent colocalization. The results revealed that there was a nuclear colocalization of HOXB7 and Ku70/Ku80/DNA‐PKcs, which became more intensive after exposure to cisplatin (Fig 6c). Above of all, it hints that HOXB7 may play a role in NHEJ to confer cisplatin resistance via interaction with Ku70/Ku80/DNA‐PKcs.

### Disrupting HOXB7 function sensitizes ESCC cells to cisplatin

**Figure 7:**
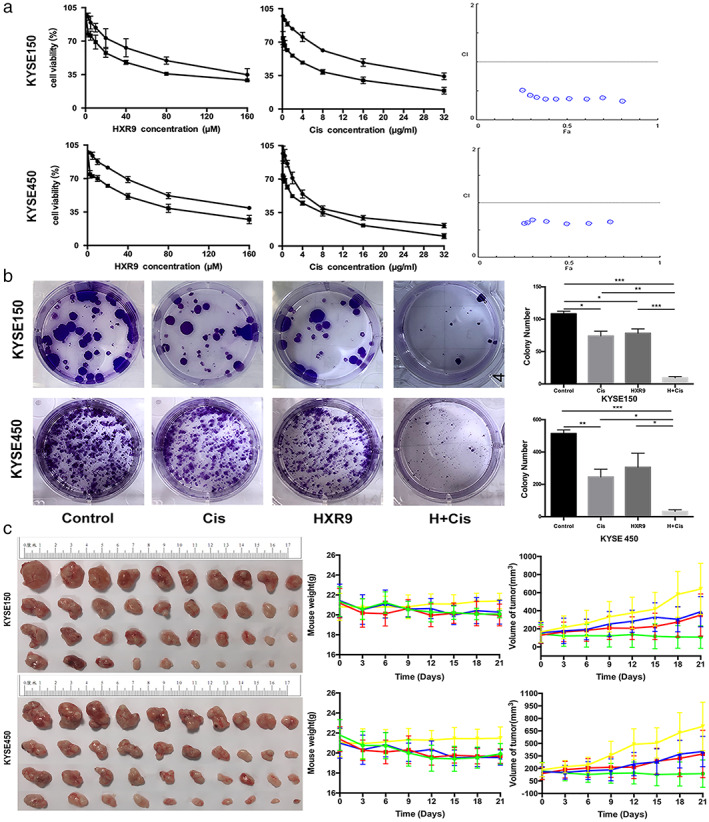
HXR9, a competitive inhibitor to disrupt HOX function, sensitizes ESCC cells to cisplatin. (**a**) Combination of HXR9 and cisplatin has a significant synergistic effect on cell viability. Cells were treated with a gradient dose of HXR9 (2.5, 5, 10, 20, 40, 80 and 160 μM) or combined with the indicated dose of cisplatin (2 μg/mL for KYSE150 and 1 μg/mL for KYSE450), as well as a gradient dose of cisplatin (0.25, 0.5, 1, 2, 4, 8, 16 and 32 μg/mL) or combination of indicated doses of HXR9 (10 μM) for 24 hours and cell viability was assessed using CCK8 assay. CI value was calculated according to the Chou‐Talalay median effect principle. The CI value ≤0.9 stands for the synergistic effect. This experiment was performed thrice in triplicates. (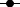) HXR9, and (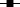) FixedCis+HXR9, and (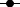) Cis (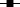) Fixed HXR9+Cis. (**b**) Combination of HXR9(10 μM) and cisplatin (2 μg/mL for KYSE150 and 1 μg/mL for KYSE450) reduced colony formation compared to monotherapy. This experiment was performed thrice in triplicates. (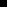) Control, (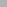) Cis, (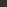) HXR9, and (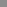) H+Cis. (**c**) HXR9 potentiated cisplatin efficacy in ESCC xenografts, and the combination causes rapid regression. Nude mice bearing KYSE150 or KYSE450 were randomly grouped and treated with 3 mg/kg cisplatin on days 1，7，14 via intravenous injection or initial dose of 100 mg/kg HXR9 followed by a subsequent dose of 10 mg/kg on day 14 via intratumoral injection or combination of the two reagents. The maximum and minimum tumors in every group were not included in analysis to deal with outliers. Tumor growth curves indicated that combination of HXR9 and cisplatin retarded the tumor growth significantly when compared with the single drug. However, the mice with combination treatment did not suffer more weight loss. (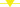) Control, (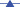) Cis, (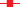) HXR9, and (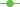) H+Cis.

**Figure 8:**
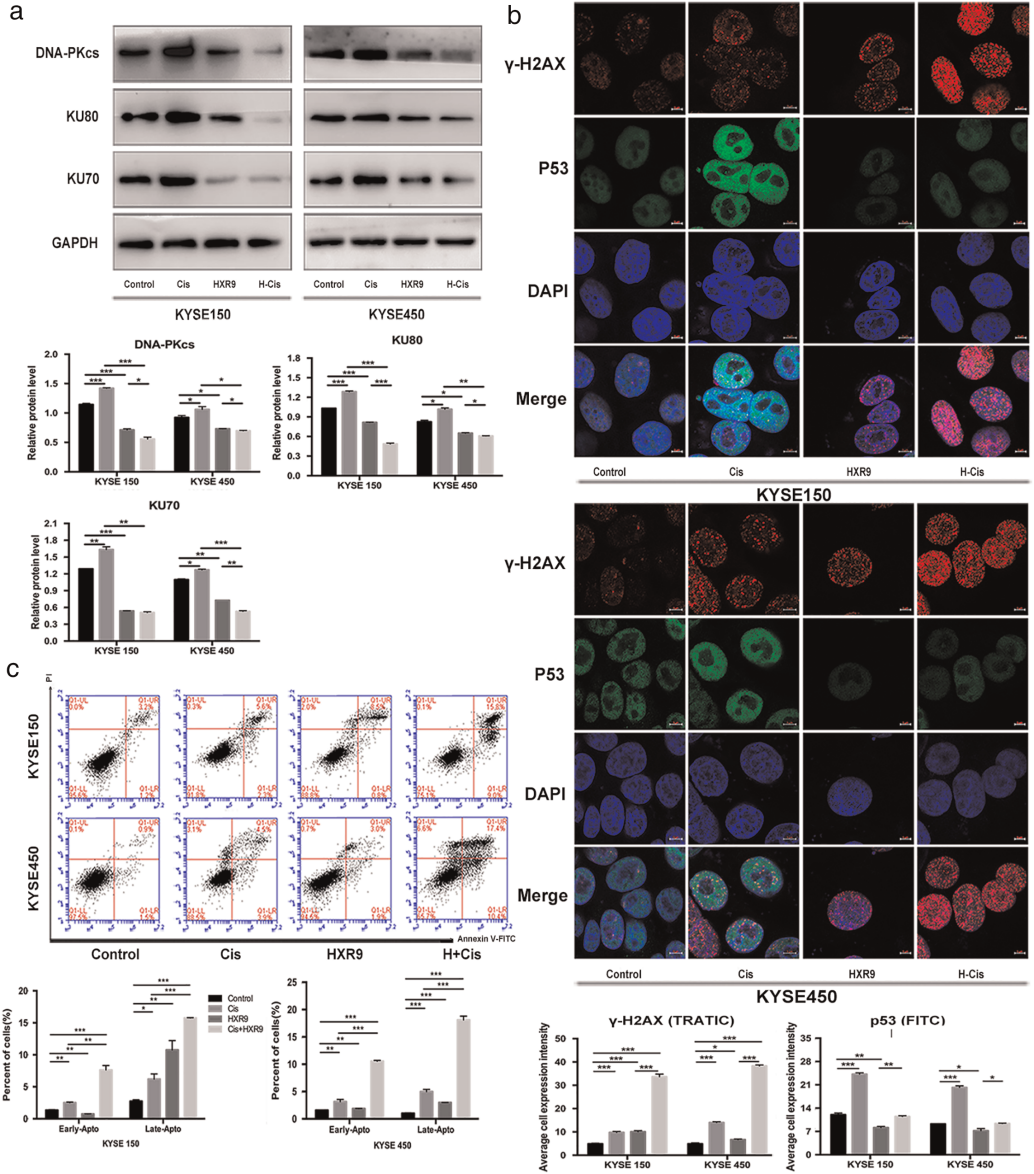
HXR9 reinforced the effects of cisplatin to induce DNA damage and cell apoptosis. (**a**) Ku70, Ku80, DNA‐PKcs expression was downregulated in HXR9‐treated cells. Combination of HXR9(10 μM) and cisplatin (2 μg/mL for KYSE150 and 1 μg/mL for KYSE450) significantly declined Ku70, Ku80, DNA‐PKcs expression compared to single agent. (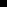) Control, (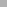) Cis, (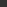) HXR9, and (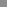) H+Cis. (**b**) HXR9 reinforced the effects of cisplatin to induce DNA damage as presented by stronger γ‐H2AX fluorescence intensity. However, HXR9 weakened the effect of cisplatin to activate DNA damage response as presented by attenuated P53 fluorescence intensity. (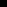) Control, (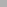) Cis, (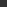) HXR9, and (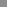) H+Cis. (**c**) Combination of HXR9 and cisplatin induced more cell apoptosis. Cells were treated with the indicated concentration of cisplatin (2 μg/mL for KYSE150 and 1 μg/mL for KYSE450), HXR9 (10 μM) or combination of HXR9(10 μM) and cisplatin (2 μg/mL for KYSE150 and 1 μg/mL for KYSE450) for 24 hours, and then underwent flow cytometry analysis by staining with Annexin V/PE. This experiment was performed thrice in triplicates.

As discussed above, HOXB7 is involved in DNA damage repair through interaction with Ku70/Ku80/DNA‐PKcs to mediate cisplatin resistance, and knockdown of HOXB7 increases chemosensitivity through cell cycle arrest and downregulates expression of Ku70/Ku80/DNA‐PKcs. It may provide a foundation for exploring and application of targeting HOXB7 as specific combination therapy. HXR9, a synthetic peptide, was designed as a competitive inhibitor to disrupt the interaction of HOX proteins with their cofactors PBX, which is required for HOX function and modifies its DNA binding specificity and affinity. Extensive researches have demonstrated that HXR9 causes apoptosis and inhibits tumor survival in a variety of tumor types, including ESCC.^23^, ^24^, ^25^, ^26^, ^27^, ^28^ To validate the hypothesis that HXR9 enhances the efficacy of cisplatin in ESCC cells, we performed a CCK‐8 assay and clonal formation assay to evaluate the cell viability after exposure to cisplatin, or HXR9, or the combination of cisplatin with HXR9 and to determine the effect of these treatments on tumor growth in ESCC mouse xenograft models. The results showed that a combination of HXR9 and cisplatin synergistically inhibited cell viability (Fig 6a,b). The growth of tumors treated with combination therapy was significantly slower than that of tumors treated with cisplatin or HXR9 alone. On day 21, the TGI (tumor growth inhibition) of KYSE150 xenograft tumor was 39.69% (cisplatin‐alone group), 40.38% (HXR9‐alone group) and 88.68% (combination group), and on day 21, the TGI of KYSE450 xenograft tumor was 37.56% (cisplatin‐alone group), 38.66% (HXR9‐alone group) and 84.36% (combination group), respectively (Fig 7c, Table S4). However, bodyweight in mice treated with combination therapy was not significantly lost, which indicated that it could be a safe therapeutic strategy. Besides, it was indicated that Ku70, Ku80, DNA‐PKcs expression was downregulated in HXR9‐treated cells, consistent with the effect of HOXB7 knockdown, but HOXB7 expression was not affected. Moreover, HXR9 undermined the expression of Ku70, Ku80, and DNA‐PKcs induced by cisplatin (Fig 8a), and HXR9 reinforced the effects of cisplatin to induce DNA damage and cell apoptosis. Specifically, γ‐H2AX fluorescence intensity was stronger upon exposure to combination therapy than monotherapy. Whereas, p53 fluorescence intensity enhancement upon exposure to cisplatin was weakened by HXR9 (Fig 8b), suggesting the DNA damage response was impaired by combination treatment. Furthermore, we observed dramatically increased cell apoptosis in cells with combination treatment (Fig 8c). Thus, HXR9 may be able to potentiate therapy response to cisplatin by disrupting HOXB7 function.

## Discussion

Platinum‐based combination remains the first line therapy for a number of solid tumors including ESCC. However, it is necessary to develop new treatment strategies and approaches in ESCC due to the development of platinum resistance. Exploring and identification of specific gene alteration predicting or mediating response to platinum is the key to consequentially developing new strategies to improve the effects.

HOX family genes are classic developmental regulators, some of which have been demonstrated to be abnormally expressed in a number of types of cancer and are involved in tumor cell proliferation, differentiation and apoptosis. It is commonly considered that dysregulated HOX genes in cancer cells are tumor modulators.^5^ Recently, more attention has been paid to the association between HOX genes and response to chemotherapy agents, such as drugs with DNA damage‐inducing properties. High expression of HOXA10 was predictive of resistance of temozolomide treatment in glioblastoma through regulation of the homologous recombinant DNA repair pathway.^10^ Increased HOXC6 expression predicted chemotherapy sensitivity in patients with ESCC.^29^ HOXA13 expression increased cisplatin resistance in ESCC cells by promoting EMT.^30^ HOXA1 might be able to act as a predictive marker of chemosensitivity. Downregulation of HOXA1 was associated with chemoresistance to cisplatin, adriamycin, and etoposide in small cell lung cancer.^11^ HOXB7 was found to simulate DNA damage repair and confer chemoresistance through interacting with Ku70, Ku80, DNA‐PKcs in colon cancer, which was involved in DSB repair.^16^ These studies provide a novel mechanism of chemoresistance mediated by HOX, suggesting that it may be a candidate target for developing therapeutic strategy to overcome drug resistance.

In our previous study, we demonstrated that HOXB7 was overexpressed in ESCC tissues to promote oncogenic properties and was predictive of a poor prognosis in ESCC.^15^ Moreover, other studies have also confirmed this.^31^, ^32^ However, to our knowledge, whether HOXB7 is correlated with chemoresistance in ESCC has not previously been addressed. In the present study, high expression of HOXB7 was determined to be associated with cisplatin resistance in ESCC cells and chemotherapy efficacy of ESCC patients. The patients with higher HOXB7 had worse TRG and outcome. Firstly, TRG is currently the standard pathological indicators of neoadjuvant chemotherapy responsiveness. Secondly, here we use the patient's long‐term survival in purpose to explain the efficacy of chemotherapy indirectly. Because we believe that the difference in long‐term efficacy of patients may be partly due to the different levels of HOXB7 expression. Therefore, prognosis of patients was a surrogate indicator for evaluating chemotherapy efficacy. We then further revealed that downregulation of HOXB7 enhanced sensitivity to cisplatin. The cell viability was significantly decreased in HOXB7‐knockdown cells upon exposure to cisplatin, and cell apoptosis was increased. We attempted to conduct the overexpression assay of HOXB7 to make the study more solid, but the western blotting results showed that the level of HOXB7 overexpression in transfected cells was not obvious because of the high background expression level.

It is well known that DNA damage repair is an important chemoresistance mechanism for cisplatin. Thus, we attempted to explore the contribution of HOXB7 to DNA damage repair to reveal the possible mechanism of its resistance phenotype. GST pulldown assay and coimmunoprecipitation unveiled the interaction of HOXB7 and NHEJ components Ku70, Ku80, and DNA‐PKcs in ESCC cells. Moreover, colocalization of HOXB7 and Ku70, Ku80, DNA‐PKcs was observed in the nucleus, implying that HOXB7 was involved in NHEJ repair pathway in association with Ku70/Ku80/DNA‐PKcs. However, the NHEJ repair pathway is error‐prone and could introduce potentially deleterious mutations and chromosomal abnormalities, which initiate tumorigenesis.^33^, ^34^ Therefore, it provides further evidence of HOXB7 functioning as a pro‐oncogenic gene. We then attempted to investigate the possible mechanisms through which HOXB7 downregulation increases chemosensitivity. The results showed that downregulation of HOXB7 induced cell cycle arrest in S phase upon cisplatin treatment. It was found that cytotoxicity is increased with exposure to cisplatin during S phase,^21^ thus, one reason of the sensitive phenotype in ESCC cells was that knockdown of HOXB7 increased S phase arrest. Another reason was that knockdown of HOXB7 decreased Ku70, Ku80 and DNA‐PKcs expression upon exposure to cisplatin, which impaired the DNA damage repair activity. Considering the role of a transcription factor, HOXB7 also involved in the transcriptional regulation of Ku70, Ku80 and DNA‐PKcs except for interaction with them, suggesting that it is not excluded transcriptional regulation of DNA damage repair genes as a possible mechanism. Hence, we identified high HOXB7 expression as a novel pathway of intrinsic resistance to cisplatin in ESCC. However, due to the general difficulty in developing effective small molecule inhibitors against transcription factors, HOX genes have not been viewed as classic therapy targets. In this study, a synthetic peptide known as HXR9, which was demonstrated to disrupt HOXB7 function by blocking HOX/PBX dimer formation and induce cancer cell apoptosis in ESCC,^28^ was utilized to inhibit HOXB7 and to determine if it had a synergistic effect to improve cisplatin chemosensitivity in ESCC cells. The results showed that combination of HXR9 and cisplatin had a synthetic lethality in vitro and in vivo. Regarding the safety of the peptide HXR9, we found that it was not cytotoxic to the nonmalignant esophagus epithelial cell line HEEC and bodyweight loss in mice treated with HXR9 was not significantly greater than the control.^28^ Meanwhile, the combination of HXR9 with cisplatin did not lead to significant weight loss in mice. In addition, previous studies have indicated that there is a rapid accumulation of HXR9 in the first few hours and it then remains stable beyond 24 hours and local delivery of HXR9 into tumors in mice has not resulted in a local inflammatory response.^24^, ^26^ Therefore, intratumor delivery of HXR9 may be a feasible approach.

Moreover, it was found that disruption of HOXB7/PBX by HXR9 caused transcription alteration of genes such as MPL, IL‐15, IL‐23A and IL‐24, which encode cytokines involved in regulating the activation of JAK‐STAT signaling pathway or induction of apoptosis in our previous study.^28^ It suggested that HXR9 may disrupt HOX/PBX functions through modifying cyctokine‐JAK‐STAT pathway activation to sensitize ESCC cells to cisplatin. However, the underlying exact mechanism is to be investigated in the future.

## Conclusion

In conclusion, our study demonstrated that high HOXB7 expression confers chemoresistance in ESCC cells through interaction with Ku70, Ku80, DNA‐PKcs and inducing cell cycle arrest, and downregulating its expression or disrupting its function enhances chemosensitivity. Therefore, we suggest that HOXB7 could be considered as a biomarker for evaluating chemoresistance and targeting HOXB7 by HXR9 may be a new therapeutic strategy to improve the effect of cisplatin.

## Disclosure

The contents of this manuscript have not been published or under consideration for publication elsewhere. There are no directly related manuscripts or abstracts, published or unpublished, by any author(s) of this paper.

## Competing interests

There are no conflicts of interest.

## Supporting information


**Figure S1.** The knockdown efficiency of HOXB7 determined by Western Blot
**Table S1.** Association between HOXB7 expression and clinic‐pathological characteristics in the study cohort (*n*=143)
**Table S2.** Association of HOXB7 expression in postoperation tumor tissues and tumor regression grade (TRG) (*n*=143)
