# Supplementary material for: HOXB7 mediates cisplatin resistance in esophageal squamous cell carcinoma through involvement of DNA damage repair
Source: Thorac Cancer. 2019 Sep 30;11(11):3071–85. doi: 10.1111/1759-7714.13142 (PMC7606015; doi:10.1111/1759-7714.13142)
Supplement: Supplementary file 1 — Figure S1. The knockdown efficiency of HOXB7 determined by Western Blot Table S1. Association between HOXB7 expression and clinic‐pathological characteristics in the study cohort (n=143) Table S2. Association of HOXB7 expression in postoperation tumor tissues and tumor regression grade (TRG) (n=143) [file TCA-11-3071-s001.docx]

Figure S1. The knockdown efficiency of HOXB7 determined by Western Blot.


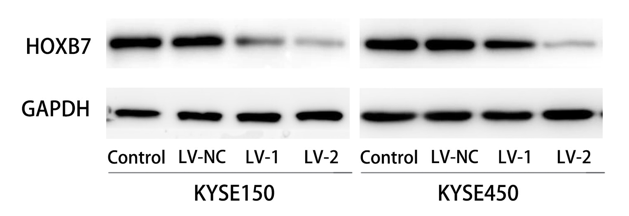


ESCC cell lines KYSE150 and KYSE450 were transfected with lentivirus LV3-HOXB7-1(5’-GCCGAGTTCCTTCAACATGCA-3’), LV3-HOXB7-2(5’-ACCTGTTCTGTAGCTTTCTGG-3’) and control virus strain LV3-NC(5’-TTCTCCGAACGTGTCACGT-3’), and the knockdown efficiency was identified by Western Blotting. The LV3-HOXB7-2 strain was selected for subsequent experiments and named KYSE150-HOXB7i and KYSE 450-HOXB7i.

Table S1. Association between HOXB7 expression and clinic-pathological characteristics in the study cohort (n=143)

| item | HOXB7 | | *P value* |
| --- | --- | --- | --- |
|  | high-expression | low-expression |  |
| Age(years) |  |  | 0.684 |
| ≤60 | 43(51.8%) | 40(48.2%) |  |
| >60 | 29(48.3%) | 31(51.7%) |  |
| Gender |  |  | 0.032 |
| Male | 59(55.7%) | 47(44.3%) |  |
| Female | 13(35.1%) | 24(64.9%) |  |
| Tumor Location |  |  | 0.634 |
| Cervical | 11(55.0%) | 9(45.0%) |  |
| Upper thoracic | 20(52.6%) | 18(47.4%) |  |
| Middle thoracic | 23(46.9%) | 26(53.1%) |  |
| Lower thoracic | 18(50.0%) | 18(50.0%) |  |
| Pathological T stage |  |  | 0.169 |
| T0/1 | 17(47.2%) | 19(52.8%) |  |
| T2 | 13(37.1%) | 22(62.9%) |  |
| T3 | 42(58.3%) | 30(41.7%) |  |
| Pathological N stage |  |  | 0.014 |
| N0 | 34(41.5%) | 48(58.5%) |  |
| N+ | 38(62.3%) | 23(37.7%) |  |

Table S2. Association of HOXB7 expression in postoperation tumor tissues and tumor regression grade (TRG) (n=143)

| Item | TRG No(%)  No.(%) | | P value |
| --- | --- | --- | --- |
|  | TRG1/2 | TRG3/4 |  |
| HOXB7 expression  (endoscope) |  |  |  |
| Low | 27(65.9%) | 44(43.1%) | 0.014 |
| High | 14(34.1%) | 58(56.9%) |  |

Table S3. Independent predictors of overall survival time in multivariate analysis (n=143)

| item | Hazard ratio | 95% CI | P value |
| --- | --- | --- | --- |
| pN |  |  | 0.006 |
| N- vs. N+ | 0.480 | 0.285-0.810 | 0.006 |
| HOXB7 expression |  |  | 0.000 |
| High vs. Low | 0.386 | 0.238-0.627 | 0.000 |

Table S4. Tumor growth inhibition of different treatment

| Item | Volume^a^ (mm3)  (Mean±SD) | | Volume^b^ (mm3)(Mean±SD) | | TGI (%) | | P | |
| --- | --- | --- | --- | --- | --- | --- | --- | --- |
|  | KYSE150 | KYSE450 | KYSE150 | KYSE450 | KYSE150 | KYSE450 | KYSE150 | KYSE450 |
| Control | 165.32±115.17 | 184.51±82.56 | 640.47±282.35 | 704.22±289.82 | - | - | - | - |
| Cis | 155.81±112.45 | 160.43±59.87 | 389.20±167.20 | 403.55±182.03 | 39.69 | 37.56 | 0.033 | <0.001 |
| HXR9 | 130.13±93.15 | 139.86±74.09 | 352.56±232.96 | 373.18±284.45 | 40.38 | 38.66 | 0.017 | 0.001 |
| HXR9+Cis | 153.43±104.32 | 169.64±101.89 | 108.10±124.05 | 137.52±163.82 | 88.68 | 84.36 | <0.001 | <0.001 |

1. The mean initial volume of tumors at treatment
2. This indicates the mean volume of tumors at the resection
